# Molecular Dynamics Simulation on the Effect of Self-Resistance Electric Heating on Carbon Fiber Surface Chemical Properties and Fiber/PP Interfacial Behavior

**DOI:** 10.3390/polym14051043

**Published:** 2022-03-05

**Authors:** Qingzhu He, Jiaqing Liu, Muhan Zhang, Zhanyu Zhai, Bingyan Jiang

**Affiliations:** 1 College of Mechanical and Electrical Engineering, Central South University, Changsha 410083, China; qingzhuhe@csu.edu.cn (Q.H.); 203711065@csu.edu.cn (J.L.); 183811004@csu.edu.cn (M.Z.); jby@csu.edu.cn (B.J.); 2 State Key Laboratory of High Performance and Complex Manufacturing, Central South University, Changsha 410083, China

**Keywords:** self-resistance electric heating, interfacial properties, molecular dynamics simulation

## Abstract

Carbon fiber-reinforced thermoplastic (CFRT) composites have been dramatically employed in the automotive field on account of their superior performances, such as being light weight and high-strength. Self-resistance electric (SRE) heating provides a solution to the problem of high energy consumption in the conventional process of CFRT composites. The effect of SRE heating on the surface chemical properties of carbon fiber (CF) was investigated by X-ray photoelectron spectroscopy (XPS). XPS analysis suggests that the C-O-C epoxy group, the CF surface, would be degraded after SRE heating with strong current intensity, while there are weak changes in the content of -C-OH, -C-O-C-, -C-NH_2_ and -COOH groups with current intensity. The interfacial bonding properties and the radial distribution function (RDF) of CF–PP interfaces were carried out by molecular dynamics (MD) simulation. The simulation results show that the adhesion between the PP and the E44 sizing agent is weaker than that between CF and PP. There are no interaction modes between the PP and E44 sizing agent except van der Waals and electrostatic adsorption. The presence of the E44 sizing agent does not change the bonding mechanism at the interface of CF/PP.

## 1. Introduction

The extensive employment of fiber-reinforced polymer materials, for instance, carbon fiber-reinforced plastic (CFRP), in the automobile field has brought about the decrease in deadweight of car and the reduction of carbon dioxide emissions [1,2,3]. Compared with thermosetting materials, carbon fiber-reinforced thermoplastics (CFRTs) have attracted considerable attention due to their excellent properties, including high tenacity, excellent impact strength and damage tolerance, short molding cycle, high productivity and retrievability [4,5,6].

With respect to the traditional processing technology of continuous carbon fiber-reinforced thermoplastics (cCFRTs), the main methods in current include dual-belt continuous molding technology [7], commingled yarn impregnation technique [8], film stacking method [9], direct injection-pultrusion technology [10]. Nevertheless, due to high melting point and viscosity of thermoplastic resin, and the heating methods of external thermal source, the traditional molding methods mentioned above are excessively high energy consumption, low efficiency and even poor impregnation degree [11]. Self-resistance electric (SRE) heating [11,12,13], which conducts current directly to the carbon fiber (CF) and heats the surrounding resin by the joule heat generated, provides an effective way to fabricate cCFRTs. 

CFs are exposed to electric–thermal load when forming cCFRTs by SRE heating. It is demonstrated that both chemical composition and active atom content on CF surface are affected by thermal load [14,15,16]. Up to now, some scholars have put effort into researching the effect of heat treatment on the surface characteristics of CF and the interfacial strength of the resulting composites. Li et al. [17] studied the effect of oven heating treatment (temperature range 280–370 °C) on the surface chemistry characteristics of CF and the interfacial properties of carbon fiber- reinforced polyether ether ketone (PEEK) composites. The results showed that after preheating treatment, the content of activated carbon atoms decreases, but the interfacial shear strength between carbon fiber and peek increases. Whereas, the interfacial shear strength (IFSS) between the CF and PEEK matrix increases, which is due to the difficulty of forming a chemical bond between peek matrix and sizing agent. It is attributed that the sizing agents of CF incompatible with PEEK are removed by preheating treatment. Dai et al. [18] researched the effect of heat treatment on the interfacial adhesion between fiber and epoxy resin. The results showed that with the increase of heat treatment temperature (150–200 °C), the contents of hydroxyl and activated carbon atoms on the CF surface decrease, resulting in the decrease of IFSS. Some researchers have explored the influence of electric current on the surface physicochemical properties of CFs. It is found that electric current significantly changes the micromorphology as well as surface functional groups of CFs [14]. Li et al. [19] characterized the mechanism of the effect of current on the interfacial properties of CFRP through a series of experiments. They reported that as the current intensity increases, the shear strength of the CF/epoxy resin interface is firstly enhanced and then weakened, which is resulted from the change of physical and chemical properties of the CF surface. Notwithstanding, their research focused on thermosetting composites. Obviously, there is a different bonding mechanism between fiber and matrix in thermoplastic composites, while there are few published reports about the effect of SRE heating on chemical properties of CFs surface and the interfacial properties of thermoplastic composites. 

In general, the formation of the fiber/matrix interface involves complex physical and chemical changes [20,21]. Hence, it is hard to reveal the micro interface adhesion mechanism of the CF/matrix interface by traditional experimental methods. Not only has molecular dynamics (MD) simulation served as a bridge from microcosmic to macroscopic, but also it is able to emulate the conditions which the conventional methods cannot achieve. Wang [22] et al. analyzed the interface bonding mechanism between epoxy resin and CF through MD simulation. They found that the shear and adsorption behaviors of the CF/epoxy resin interface are significantly related to the number of functional groups. Stoffels et al. [23] applied MD simulation to the study of interface failure mechanism of fiber-reinforced composites, trying to explore the influence of water absorption of epoxy resin on the interface behavior of glass fiber-reinforced thermosetting resin composites. The simulation results demonstrated that the work of adhesion between glass fiber and sizing agent is extremely sensitive to the water absorption behavior of epoxy resin. Niuchi et al. [24] investigated the influence of chemical changes of the CF surface on the interfacial stress and fracture energy of the CF/phenolic resin interface by MD simulation. The research findings indicated that the interfacial strength between CF and phenolic resin is closely associated with the proportion of trifluoromethyl (CF_3_) on the CF surface. Even so, the understanding of the bonding mechanism between thermoplastic matrix and CF with different surface chemical compositions is still somewhat limited or lacking.

Against this background, the aims of this paper were to investigate the effect of SRE heating on the chemical properties of CF surface and to explore the interfacial bonding mechanism of the resulting composites under such conditions through MD simulations. Firstly, the changes of chemical properties on the CF surface after SRE heating with different current magnitudes were detected by X-ray photoelectron spectroscopy (XPS). Based on the results of XPS analysis, five kinds of CF models were established to evaluate the interfacial properties between polypropylene (PP) and CF with different surface chemical properties by Materials Studio 7.0 software (Accelrys Software lnc., San Diego, CA, USA). The equilibration procedure and the uniaxial tensile deformation were carried out by MD simulation. Firstly, the impregnation quality was systematically studied by analyzing the density distribution of the PP along the height direction. Then, interfacial bonding energy was calculated and Radial Distribution Functions (RDF) analysis was performed to explore the bond mechanism of the CF–PP interface. Finally, the uniaxial tensile simulation was carried out to study the interfacial strength and failure mechanism of the CF–PP interface.

## 2. Materials and Methods

### 2.1. Material and Equipment

**Figure 1:**
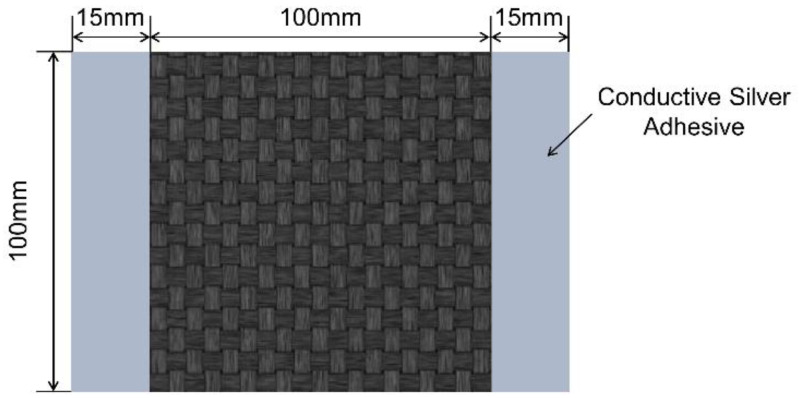
Geometric dimension of carbon fiber fabric sample and coating position of conductive silver adhesive.

**Figure 2:**
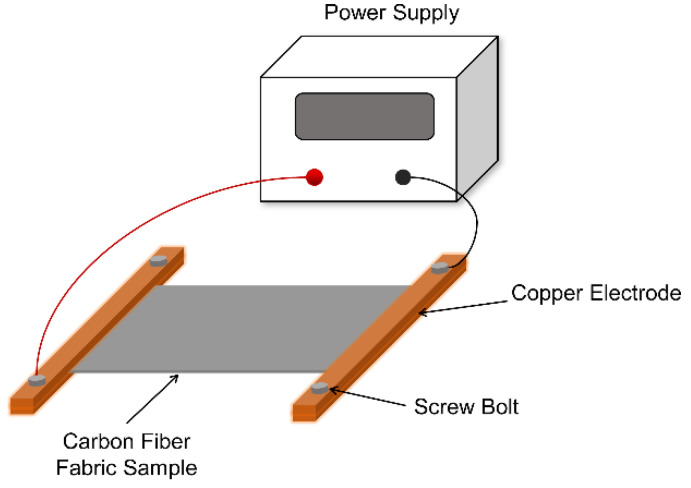
The sketch map of SRE heating platform for carbon fiber fabric.

In this study, polyacrylonitrile (PAN)-based carbon fiber fabric (T300B-CO6343B), purchased from TORAY Company, Japan, was employed as the raw material. In order to reduce the contact resistance between the carbon fiber fabric and the copper electrodes, a thin layer of conductive silver adhesive (DS-5510, D-MAX Technology Co., Ltd., Shenzhen, China) with a width of 15 mm was coated on both ends of the carbon fiber fabric sample. The size of the carbon fiber fabric sample and the coating position of conductive silver adhesive are shown in Figure 1. The schematic diagram of the SRE heating platform for carbon fiber fabric is displayed in Figure 2. The power supply used in this study is KEITHLEY 2260B-30-108 (Tektronix Inc co., LTD, Beaverton, OR, USA), with maximum voltage of 30 V. In the experiment, the current loads applied to the carbon fiber fabric were 0 A, 12 A, 16 A, 20 A and 24 A, respectively, and the electrify time was 240 s. The infrared thermal imager (ImageIR 8300, InfraTec GmbH, Dresden, Germany) was used to monitor the temperature change of carbon fiber fabric under SRE heating. Figure S1 shows the temperature field distribution of carbon fiber fabric under SRE heating with the current intensity of 12 A, 16 A, 20 A and 24 A. As seen, the surface temperature of carbon fiber fabrics under the electric intensity of 12 A, 16 A, 20 A and 24 A at steady stage are 158 ± 2.9 °C, 212 ± 3.2 °C, 270 ± 4.9 °C and 332 ± 5.4 °C, respectively.

### 2.2. Characterization

X-ray photoelectron spectroscopy (XPS; ESCALAB 250 Xi, Thermo Fisher Scientific Inc., Waltham, MA, USA) was used to analyze the change of chemical composition on the CF surface after SRE heating with different current intensity. The tests were carried out with a monochromatic Al Kα X-ray radiation source, which has a spot size of 500 μm, operating at a chamber pressure of 5.0 × 10^−10^ mbar. Spectra were analyzed with Avantage software (5.52, Thermo Fisher Scientific Inc., Waltham, MA, USA) by deducting a Shirley background and fitting Gaussian–Lorentzian function.

### 2.3. Experimental Results

**Figure 3:**
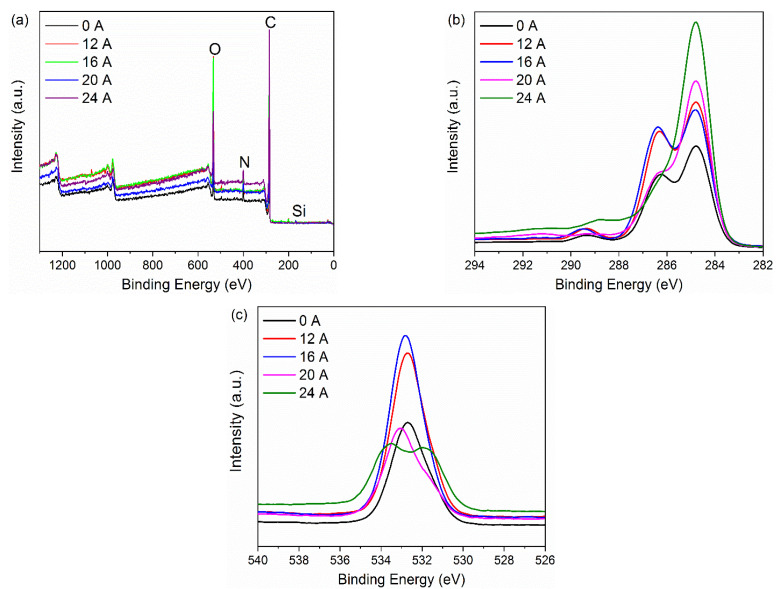
XPS spectra of the samples treated with different current intensities: (**a**) full XPS spectra, (**b**) the C 1s spectra, (**c**) the O 1s spectra.

Figure 3 displays the XPS scan spectra and chemical element content of the CF surface. From the full-scan spectrum of XPS spectra on the CF surface (Figure 3a), it is apparent that there are mainly four elements, namely, C, O, N and Si, on the CF surface, regardless of the current intensity. As can be seen from Figure 3b,c, the peak profile and intensity of C 1s and O 1s narrow scan spectra change with the current intensity. In particular, at the current intensity of 24 A, there is a change from a double peak to a prominent single peak at the C 1s spectrum, while the O 1s spectrum appears differently from a single peak to a double peak; moreover, the peak intensity decreases sharply. It indicates that the functional groups and the chemical element content change dramatically after applying a stronger current intensity. In addition, it can be seen from Table 1 that the O/C atomic ratio shows no obvious change in the range of current intensity from 0 A to 16 A. However, after 16 A, the O/C atomic ratio on the CF surface decreases apparently. Especially, the content of carbon element increases from 75.55% (0 A) to 82.89% (24 A), while the content of oxygen element decreases to 13.33% at 24 A. It indicates that the chemical activity of the CF surface decreases due to SRE heating at stronger current intensity, which is caused by the decomposition of the sizing agent under elevated temperatures. Similar experimental phenomena have been reported in previous studies [14].

**Figure 4:**
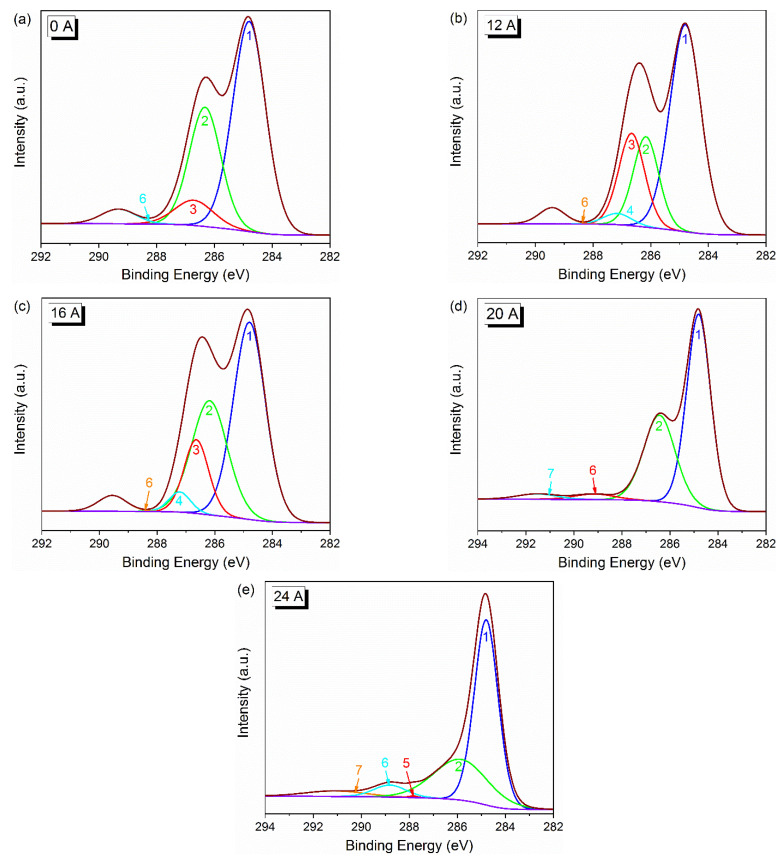
Deconvolution of C 1s XPS spectra of the CF surface: (**a**) untreated, (**b**) 12 A/240 s, (**c**) 16 A/240 s, (**d**) 20 A/240 s and (**e**) 24 A/240 s.

**Figure 5:**
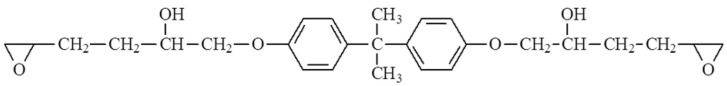
Chemical structure of E44.

The high-resolution spectra of C 1s were deconvoluted to further analyze the functional groups on the CF surface. Figure 4 shows the detailed deconvolution results of the C 1s. As seen, there are seven potential carbon-containing component with bonding energies of: 284.6 eV (-C-C-), 286.1 eV (-C-OH, -C-O-C- and -C-NH_2_), 286.6 eV (C-O-C epoxy groups), 287.2 eV (-C-OR), 287.5 eV (-C=R), 288.9 eV (-COOR, -COOH), 291.1 eV (π-π) [18,25,26]. Especially, as suggested by reference [18], the peak at 286.6 eV is attributed to an epoxy group. It can be understood that the main ingredients of sizing for T300B fiber are bisphenol A epoxy resin and amino compounds, as reported by [27]. Herein, the activated carbon atoms (carbon atoms conjunct with oxygen and nitrogen) concentration is introduced to represent the chemical reactivity of the CF surface. The relative amount of surface functional groups, the activated carbon fiber atoms concentration, and the value of B.E. are listed in Table 2. Figure 5 shows the chemical structure of E44. The C-O-C epoxy group in the third column of Table 2 refers to the epoxy group with triangular structure at both ends of E44 molecular formula, while the C-O-C in the second column is not a triangular epoxy structure. As seen, the C-O-C epoxy group on the CF surface decreases to 0 after SRE heating at 20 A, which illustrates that the epoxy sizing on the CF surface would degrade after SRE heating with stronger current intensity. The decrease in the activated carbon atom concentration can also verify this, while peak 2 and peak 6 exhibit a weak change when current intensity increases from 16 A to 20 A (or 24 A). Besides, π-π appears when the current intensity reaches more than 20 A. Because the CF precursor is composed of disordered graphite microcrystals arranged in two-dimensional order along the fiber axis, the appearance of π-π can prove that the CF precursor is exposed after the sizing agent on the CF surface is decomposed [28,29]. To sum it up, too strong of a current intensity can bring about the thermal decomposition of some sizing agents, and the reduction of oxygen content on the CF surface and the content of activated carbon atom, inhibiting the chemical activity of the CF surface.

## 3. Simulation Method

As discussed in Section 2.3, there are variations in the chemical properties on CF surfaces under SRE heating with different current intensity. MD simulation was carried out for qualitative evaluation of the influence on the interfacial properties of CF/PP composites from the changes in the chemical properties.

### 3.1. Model Construction

To build the model of the CF–PP interface, the CF and PP models were separately constructed by Materials Studio 7.0 software.

#### 3.1.1. PP Layer

**Figure 6:**
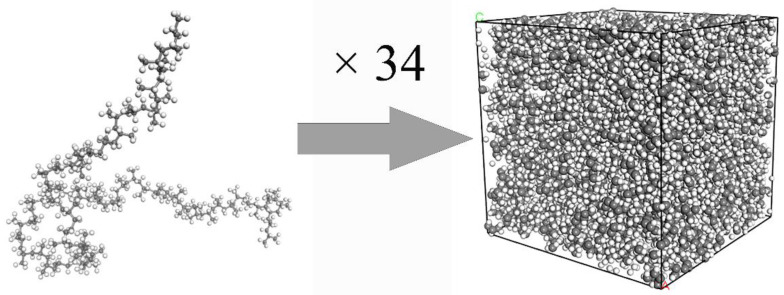
Formation of the PP layer.

As shown as Figure 6, 34 PP chains formed the PP layer in a square box with dimensions of 5.4 nm (length) × 5.2 nm (width) × 6.4 nm (height). The degree of polymerization was 70 and density was 0.92 g/cm^3^. The periodic boundary of the model was set to approximate a polymers large system.

The initial model was not stable due to the unreasonable molecular structure. Energy minimization, a subsequent annealing treatment and relaxion were used to optimize the conformation of the PP layer. The specific operations are as follows: the energy minimization was performed by using the steepest descent, conjugate gradient and Newtown methods. Iterations of every method are 10,000. Then, the annealing simulation was carried out. The initial temperature and the maximum annealing temperature were 300 K and 600 K, respectively, and the annealing cycle was 20 times. Finally, the PP layer was relaxed for 100 ps under the NVT ensemble.

#### 3.1.2. CF Layer

**Figure 7:**
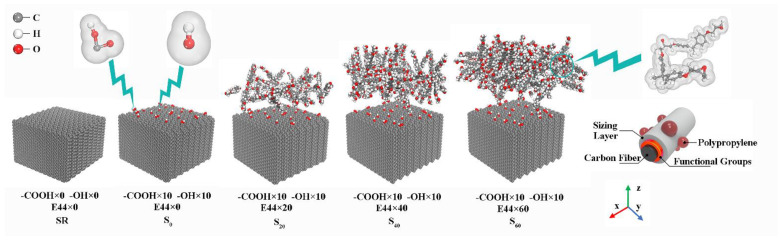
Carbon fiber models with different numbers of E44.

Same with our previous investigation [30], the CF model was created using 15 perpendicular graphene layers with a close-packed hexagonal structure and 0.34 nm spacing, which has the dimensions of 5.4 nm × 5.2 nm × 3.5 nm. As revealed by XPS, the sizing agent on the CF surface was epoxy compound. However, it is difficult to know the concrete sizing agent for CF. Here, taking the same approach with reference [31], the epoxy sizing of E44 was simply chosen for the CF surface in this paper. As shown in the XPS results, the sizing agent on the CF surface changes with current intensity in the form of epoxy group (C-O-C). To explore the possible effect on the CF–PP interface from this change, the number of E44 of different CF layers was set as 0, 20, 40 and 60, respectively, and the corresponding interface models of composites are called S_0_, S_20_, S_40_ and S_60_ for short, as shown in Figure 7. XPS results show that hydroxyl and carboxyl groups exist on the CF surface under different current intensities. However, the percentage of carbonyl group is relatively low (max.0.2%) compared with other functional groups. Besides, Uematsu et al. [32] found that the difference of 10% in the content of C=O is a minor factor in the interfacial adhesion between PA6 and CF. Furthermore, the complexity of MD model requires high computational resources. Thus, the influence of carbonyl groups was not considered in the MD simulation. According to the research of Jiao et al. [31], the surface grafting rate was set as 6.06% and ten hydroxyl and carboxyl groups were introduced on the CF surface.

### 3.2. Dynamic Simulations

#### 3.2.1. Equilibration Stage

The equilibration steps were carried out in the NPT ensemble (i.e., the number of atoms, pressure and temperature are constant), and the pressure was kept at 4 MPa, which was applied along the impregnation direction (the negative Z direction). The boundary conditions of the model box were periodic except for the free boundary conditions in the Z direction to ensure that the bonding of CF and PP can be done under injection pressure. The detailed process of the equilibration was as follows: the system was heated up to 600 K from 300 K at the rate of 2 K/ps, and dwells for 100 ps. Then, the system was cooled down to 100 K from 600 K at the rate of 2 K/ps.

#### 3.2.2. RDF Analysis

**Figure 8:**
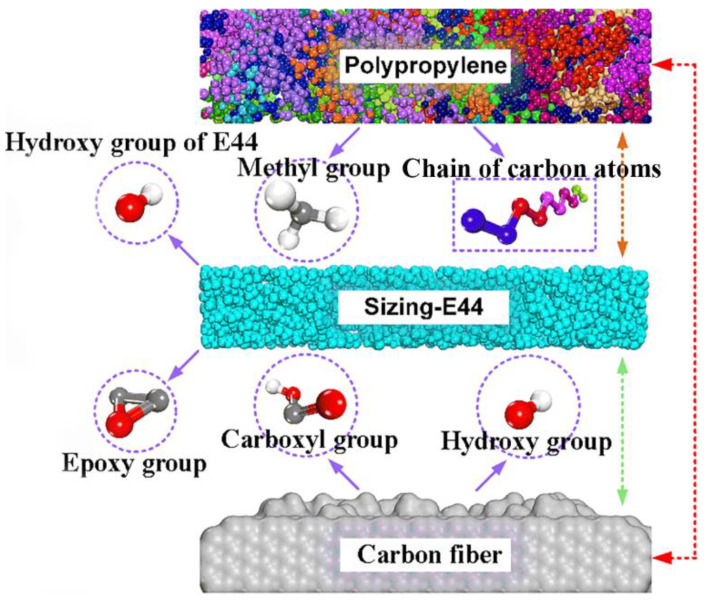
Schematic diagram of main atomic groups at the CF–PP interface.

In order to deeply understand the effects on bonding mechanism between CF and PP from chemical properties of CF surface, the radial distribution function (RDF) was used to further analyze the CF–PP interface at the last frame of the equilibration process. The RDF describes the probability density of the existence of another molecule in a system at a distance of r from any molecule. It can be expressed as [33]:

(1) $$g\left(r\right)=\frac{n\left(r\right)}{\rho_{0}V}\approx \frac{n\left(r\right)}{4\pi r^{2}\rho_{0}\delta_{r}}$$

where r is the radius from the central atom, $\delta_{r}$ is the spherical shell thickness, n(r) is the number of particles in a spherical shell and $\rho_{0}$ is number density in ideal crystals. Before calculating *g*(*r*), it is necessary to calibrate the key atomic groups on the polymer layer, sizing agent layer and CF surface, as shown in Figure 8. Hydroxy group of E44, epoxy group of E44, methyl group of PP, chain of carbon atoms of PP, carboxyl group of CF and hydroxy group of CF are referred by OH(E44), COC(E44), CH_3_(PP), CC(PP), COOH(CF) and OH(CF), respectively.

#### 3.2.3. CF–PP Interface Separation

**Figure 9:**
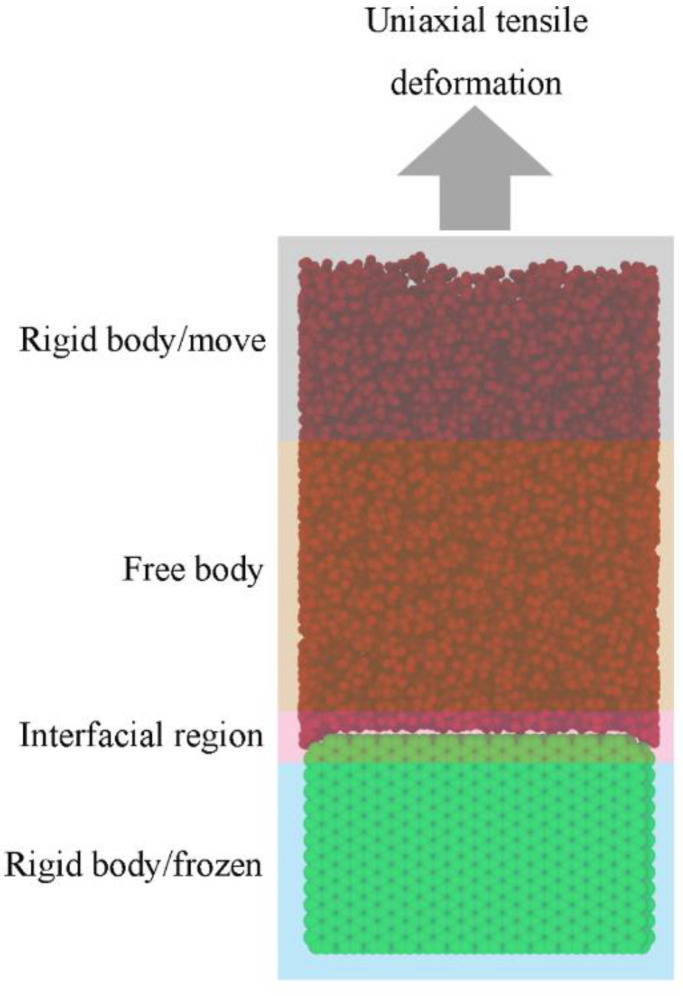
Uniaxial tensile simulation method.

After the equilibration stage was completed, the tensile deformation was carried out to study tensile mechanical properties and failure mechanism of the CF–PP interface. The method of the tensile deformation simulation is shown in Figure 9. The top layer of the PP and the CF layer were set as rigid bodies, respectively, and the atoms in other areas of the system were kept free. Under the NVT ensemble with the temperature of 100 K, the temperature of the interface separation process was controlled by using the nose Hoover thermostat. The rigid body of the top polymer was displaced continuously for 100 ps at a fixed speed of 0.1 nm/ps along the positive direction of the Z axis.

All MD simulations were performed by using Large-scale Atomic/Molecular Massively Parallel Simulator (LAMMPS) [34]. The consistent valence force field (CVFF) was used to depict the intermolecular and non-bonding interactions. The force field covers not only bond stretching, angular bending, and torsion, but also non-bond interactions, including Lennard–Jones (12–6) and Coulomb potentials; 1.25 nm was chosen as the value of the cutoff radius of MD simulations [35].

## 4. Discussion

### 4.1. Interfacial Bonding Properties of CF/PP Composites

**Figure 10:**
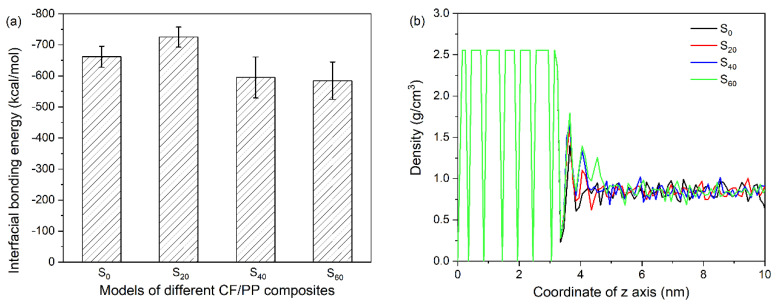
(**a**) Interfacial bonding energy and (**b**) density distribution along z axis of CF/PP composites.

Figure 10a gives the interfacial bonding energy of CF/PP composites with different numbers of E44 molecules on the CF surface. As seen, with the increase of the number of E44 molecules from 0 to 20, the interfacial interaction energy increases obviously. It can be understood that the molecular conformation formed by E44 molecules on the CF surface improves the surface roughness of CFs [36], resulting in a higher interaction energy of S_20_. When the number of the E44 molecules increases to 40 or 60, the thickness of the sizing agent is enough to block the interaction between the PP layer and CF layer. In this case, the sizing agent layer is bonding with the PP layer instead of the CF. Therefore, the interfacial interaction energy barely changes when the number of the E44 molecules is 40 or 60.

Figure 10b shows the density distribution of the PP layer along the height direction (Z axis). It can be found that the density distribution curve has an obvious oscillation trend, and the maximum amplitude appears near the surface of CF (3 nm). The first two oscillation amplitudes of each curve near the fiber surface have little difference. This phenomenon shows that the change of the number of E44 molecules does not significantly change the density of the interfacial phase, indicating that there is no strong intermolecular force between the sizing agent and the PP layer.

**Figure 11:**
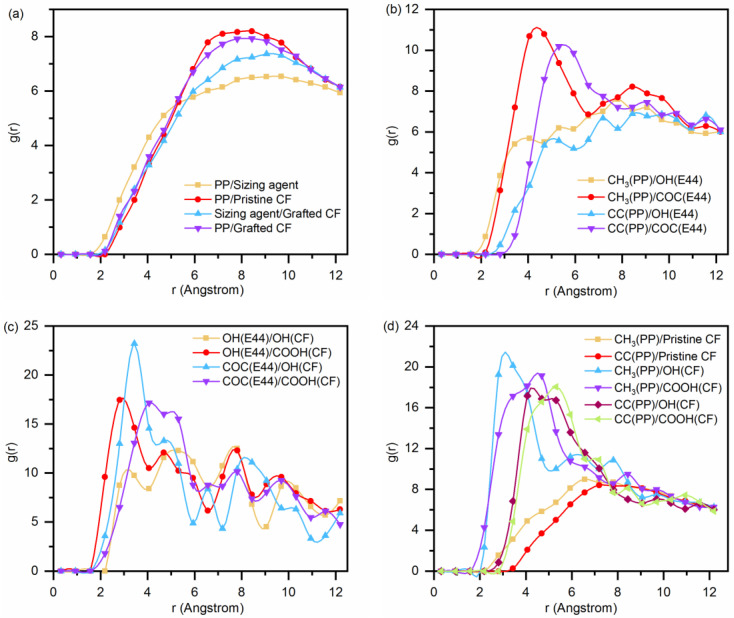
The RDFs for: (**a**) PP/sizing agent (S_60_), PP/pristine CF (SR), sizing agent/grafted CF (S_60_) and PP/grafted CF (S_0_); (**b**) the active groups of PP/sizing agent(S_60_); (**c**) sizing agent/active groups on the surface of CF (S_60_); (**d**) CH_3_(PP)/Pristine CF (SR), CC(PP)/Pristine CF (SR), CH_3_(PP)/OH(CF) (S_0_), CH_3_(PP)/COOH(CF) (S_0_), CC(PP)/OH(CF) (S_0_) and CC(PP)/COOH(CF) (S_0_).

The peak position r of RDF can directly reflect the type of intermolecular interactions. When the peak position is in the range of 0–0.20 nm, 0.20–0.31 nm and 0.31–0.50 nm, the interactions are chemical bond, hydrogen bond, van der Waals and electrostatic interaction, respectively [33]. The RDFs for SR, S_0_ and S_60_ models are shown in Figure 11. As can be seen from Figure 11a, the peak position of the PP/sizing agent curve is larger than that of the other three groups, and the value of RDF is the smallest. The larger the value of RDF at the same distance, the greater the bonding strength of the interface. It indicates that the adhesion ability between the PP and the sizing agent is the weakest, which is not as good as the interaction between PP and pristine CF. In addition, it is also observed that the RDF value of the PP/grafted CF interface is slightly higher than that of the PP/pristine CF interface. This may result from the fact that the functional groups increase the distance between the resin molecules and the carbon atoms of the fiber from the spatial position, resulting in a certain spatial obstruction [36]. The specific RDF of CH_3_(PP), OH(E44), COC and CC(PP) are shown in Figure 11b. The RDF peak positions of CH_3_/OH(E44) and CH_3_/COC are higher than those of CC/OH(E44) and CC/COC, respectively. The CH_3_(PP) is more active than CC(PP) when substances are attracted to each other. According to the peak position, the van der Waals force and electrostatic interaction maintain the attraction between the CH_3_ and the oxygen-containing functional group of sizing agent, while the CC and the oxygen-containing functional group of sizing agent are mainly weak van der Waals force. As shown in Figure 11c, peaks appear in the range of 0.20–0.30 nm of the RDF of OH(E44)/COOH(CF) and OH(E44)/COOH(CF), indicating the formation of hydrogen bonding between the OH(E44) and COOH(CF). Since the magnitude of hydrogen bonding mainly depends on the electronegativity of the groups, the electronegativity of the OH is slightly weaker than that of the COOH [36,37]. Therefore, the RDF peak position of OH (E44)/OH (CF) is weaker than that of OH(E44)/COOH (CF) within this range. Although there is no strong hydrogen bond between the COC and the grafted function groups, strong van der Waals force can be formed. It is worth noting that these curves are not unimodal structure, but multi-peak structure, which may be related to the obvious anisotropy of the interface characterized by the uneven topography of the interface. As shown in Figure 11d, the CH_3_ forms strong hydrogen bonds with OH and COOH, respectively. Strong van der Waals force and electrostatic adsorption exist between CH_3_ (COC) and OH, as well as that between CH_3_ (COC) and COOH. However, the van der Waals force and electrostatic adsorption between CH_3_ and pristine CF, as well as that between CH_3_ and pristine CF, is relatively small. In summary, there are no interaction modes between the PP and sizing agent except van der Waals and electrostatic adsorption, and the adsorption ability is not as strong as that between the PP and CF. On the whole, the presence of the sizing agent does not change the nature of the bonding mechanism at the interface of CF/PP. On the contrary, the excessively thick sizing layer weakens the interfacial bonding strength of the composites. It can conclude that the degradation of sizing agent because of SRE heating with strong current intensity does not affect the bonding between the CF and PP from nanoscale aspect. The related experimental data obtained from reference [38] also verify that. It might be that the epoxy sizing on the CF surface is not compatible with thermoplastic matrix.

### 4.2. Uniaxaial Tensile Deformation Process

**Figure 12:**
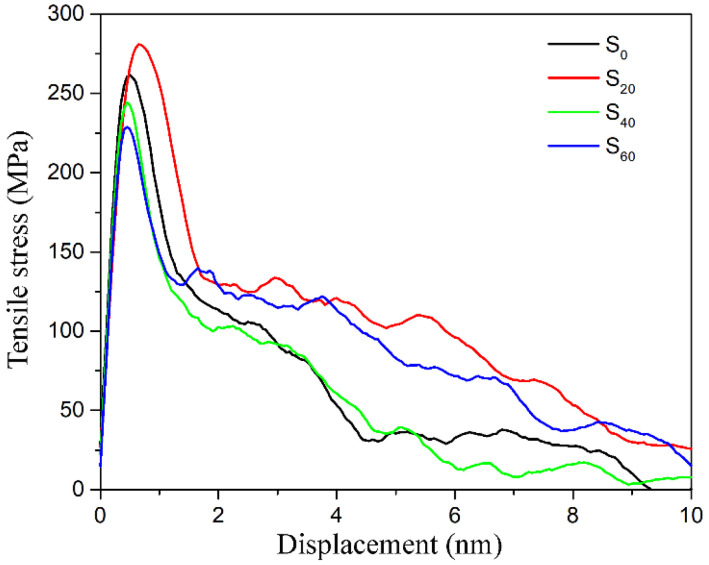
Uniaxial tensile deformation stress-displacement curves of the CF–PP interface under different number of E44.

The tensile stress-displacement curves of different models (S_i_) under uniaxial tensile loading are shown in Figure 12. The curves exhibit three classical regions during the whole tensile deformation process, namely, elastic, yield and softening stages. When the number of E44 molecules is 20, the interfacial tensile strength reaches the highest value, namely, 280.94 MPa, which is nearly 7.4% higher than that of the S_0_ model without the sizing agent and only grafting functional groups on the CF surface. When the number of E44 molecules increases to 40 and 60, the sizing agent layer covers the CF surface completely, but the interfacial tensile strength decreases, which is 244.25 MPa and 228.73 MPa, respectively. This trend is the same as that of the interfacial bonding energy, which further verifies that the sizing agent is not conducive to the interfacial property. According to the RDF analysis, the sizing agent has weaker ability to attract the PP, compared with CF. To some extent, the connection between the CF and PP is blocked by the sizing agent. However, a thin sizing agent cannot weaken the interfacial strength. On the contrary, irregular physical morphology of the sizing agent enhances the mechanical interlocking. A thick sizing agent layer can block the interaction between the CF and PP completely. Therefore, the sizing agent with appropriate thickness can increase interfacial tensile strength of CF/PP composite.

**Figure 13:**
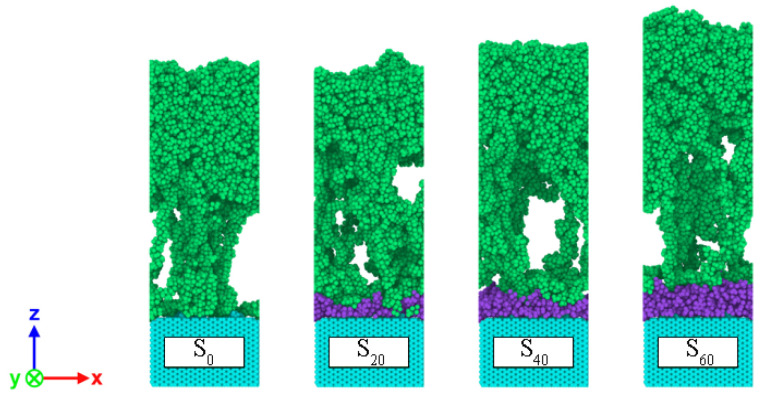
Failure modes of the S_i_ interface system during uniaxial tensile deformation simulation at 40 ps.

**Figure 14:**
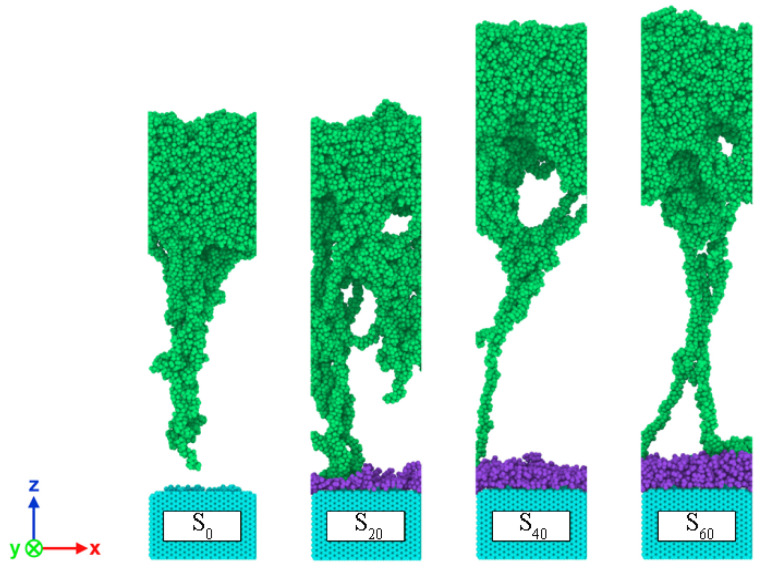
Morphology of tensile failure of the S_i_ interface system at 100 ps.

The failure mechanism of the CF–PP interface system is the competition between the CF–PP interface and PP, and the different competition pattern can create three failure modes. They are adhesive failure, cohesive and adhesive mixed failure and cohesive failure. Figure 13 and Figure 14 clearly represent the interface failure position and tensile failure morphologies. It is the cohesive and adhesive mixed failure for S_20_; the failure modes for the other three models are all adhesive failures. When the stretching lasted to the 100 ps, the tail of the PP chain of S_0_ is completely separated from the CF surface, while some PP chains of the other three models are still attached to the surface of the sizing agent. The cause of this phenomenon may be related to the rough surface of different sizing agent layer.

## 5. Conclusions

In this research, XPS tests and MD simulation were carried out to study the effect of chemical properties on the CF surface on interfacial behavior of CF/PP composites under SRE heating. The main conclusions of this work are:

(1) The XPS analyses reveal that the C-O-C epoxy group decreases to 0 after SRE heating at 20 A because of the epoxy sizing agent on the CF surface degrading at strong current intensity. It leads to a decrease in the chemical activity of the CF surface. In addition, there are weak changes in the content of -C-OH, -C-O-C-, -C-NH_2_ and -COOH groups with current intensity.
(2) There are no interaction modes between the PP and sizing agent except van der Waals and electrostatic adsorption. Moreover, the adsorption of PP molecules on the sizing agent is not as strong as that between the PP and CF. It indicates that the presence of the sizing agent does not change the nature of the bonding mechanism at the interface of CF/PP. It can be obtained that the degradation of the sizing agent because of SRE heating with strong current intensity does not affect the bonding between the CF and PP from nanoscale aspect.
(3) The interfacial tensile strength reaches the maximum when the number of E44 molecules is 20, that is, 280.94 MPa, which is nearly 7.4% higher than that when the number of E44 molecules is 0. However, when the number of E44 molecules increases to 40 and 60, the interfacial tensile strength decreases by 13.1% and 18.6%, respectively compared with that of 20 molecules of E44. This trend is corresponding with the variation trend of interfacial interaction energy.

## Figures and Tables

**Table 1:** Analysis of chemical elements on the CF surface.

**Current Intensity** **(A)**	**Element Composition (%)**				**O/C**
	C	O	N	Si	
0	75.55	20.57	3.77	0.1	0.272
12	75.01	21.01	3.79	0.19	0.280
16	74.38	21.69	3.75	0.19	0.292
20	81.9	14.79	3.27	0.04	0.181
24	82.89	13.33	3.7	0.07	0.161

**Table 2:** Relative contents of functional groups on the CF surface.

**Current** **Intensity** **(A)**	**Contribution of C 1s Components (%)**							**Activated** **Carbon Atoms Concentration** **(%)**
	-C-C-	-C-OH -C-O-C- -C-NH_2_	C-O-C epoxy groups	-C-OR	-C=O	-COOR -COOH	π-π	
	Peak 1	Peak 2	Peak 3	Peak 4	Peak 5	Peak 6	Peak 7	
	284.6 B.E./eV	286.1 B.E./eV	286.6 B.E./eV	287.2 B.E./eV	287.5 B.E./eV	288.9 B.E./eV	291.1 B.E./eV	
0	56.89	30.9	8.03	0	0	4.18	0	43.11
12	54.32	19.32	20.14	2.66	0	3.56	0	45.68
16	48.25	31.14	13.82	3.35	0	3.44	0	51.75
20	59.61	34.80	0	0	0	2.87	2.73	40.39
24	59.91	30.05	0	0	0.2	5.32	4.51	40.09

## Data Availability

Data are contained within the article.

## Supplementary Materials

The following are available online at https://www.mdpi.com/article/10.3390/polym14051043/s1, Figure S1: Temperature field distribution of carbon fiber fabric under SRE heating with different current intensity.

## References

[1] Al-Lami A., Hilmer P., Sinapius M. (2018). Eco-Efficiency Assessment of Manufacturing Carbon Fiber Reinforced Polymers (CFRP) in Aerospace Industry. Aerosp. Sci. Technol..

[2] Khalil Y.F. (2017). Eco-Efficient Lightweight Carbon-Fiber Reinforced Polymer for Environmentally Greener Commercial Aviation Industry. Sustain. Prod. Consum..

[3] Subhani M., Globa A., Al-Ameri R., Moloney J. (2017). Flexural Strengthening of LVL Beam Using CFRP. Constr. Build. Mater..

[4] Wellekötter J., Bonten C. (2020). Direct Joule Heating as a Means to Efficiently and Homogeneously Heat Thermoplastic Prepregs. Polymers.

[5] Bussetta P., Correia N. (2018). Numerical Forming of Continuous Fibre Reinforced Composite Material: A Review. Compos. Part A Appl. Sci. Manuf..

[6] Friedrich K. Carbon Fiber Reinforced Thermoplastic Composites for Future Automotive Applications. Proceedings of the VIII International Conference on “Times of Polymers and Composites”: From Aerospace to Nanotechnology.

[7] Trende A., Åström B.T., Wöginger A., Mayer C., Neitzel M. (1999). Modelling of Heat Transfer in Thermoplastic Composites Manufacturing: Double-Belt Press Lamination. Compos. Part A Appl. Sci. Manuf..

[8] Sugimata E., Ueda H., Kuriyama W., Okumura W., Kimizu M., Taka M., Mori D., Uzawa K. (2017). Formability of Braided CFRTP Cylindrical Pipe in Pipe Bending. J. Text. Eng..

[9] Guo Y. (2016). Research on Thermoplastic Composites and Its Application in the Field of Aviation. Fiber Compos..

[10] Babeau A., Comas-Cardona S., Binetruy C., Orange G. (2015). Modeling of Heat Transfer and Unsaturated Flow in Woven Fiber Reinforcements during Direct Injection-Pultrusion Process of Thermoplastic Composites. Compos. Part A Appl. Sci. Manuf..

[11] Liu S., Li Y., Shen Y., Lu Y. (2019). Mechanical Performance of Carbon Fiber/Epoxy Composites Cured by Self-Resistance Electric Heating Method. Int. J. Adv. Manuf. Technol..

[12] Zhang B., Li Y., Liu S., Shen Y., Hao X. (2021). Layered Self-resistance Electric Heating to Cure Thick Carbon Fiber Reinforced Epoxy Laminates. Polym. Compos..

[13] Wang X., Liu Y., He Q., Weng C., Zhai Z. (2021). Fabrication of Continuous Carbon Fiber Reinforced Polyamide 6 Composites by Means of Self-resistance Electric Heating. Polym. Compos..

[14] Wang Z., Zhao X., Lu P., Li N. (2016). Effect of Electric-Thermal Load on the Physical and Chemical Properties of Carbon Fiber’s Surface. Surf. Technol..

[15] Dai Z., Zhang B., Shi F., Li M., Zhang Z., Gu Y. (2012). Chemical Interaction between Carbon Fibers and Surface Sizing. J. Appl. Polym. Sci..

[16] Wu Q., Zhao R., Ma Q., Zhu J. (2018). Effects of Degree of Chemical Interaction between Carbon Fibers and Surface Sizing on Interfacial Properties of Epoxy Composites. Compos. Sci. Technol..

[17] Li N., Chen J., Liu H., Dong A., Wang K., Zhao Y. (2019). Effect of Preheat Treatment on Carbon Fiber Surface Properties and Fiber/PEEK Interfacial Behavior. Polym. Compos..

[18] Dai Z., Zhang B., Shi F., Li M., Zhang Z., Gu Y. (2011). Effect of Heat Treatment on Carbon Fiber Surface Properties and Fibers/Epoxy Interfacial Adhesion. Appl. Surf. Sci..

[19] Li N., Wang Z., Lu P. (2019). Influence of Electro-Thermal Effect on Interfacial Property of Carbon Fiber/Epoxy Resin. J. Funct. Mater..

[20] Tam L., Zhou A., Wu C. (2019). Nanomechanical Behavior of Carbon Fiber/Epoxy Interface in Hygrothermal Conditioning: A Molecular Dynamics Study. Mater. Today Commun..

[21] Xu P., Yu Y., Guo Z., Zhang X., Li G., Yang X. (2019). Evaluation of Composite Interfacial Properties Based on Carbon Fiber Surface Chemistry and Topography: Nanometer-Scale Wetting Analysis Using Molecular Dynamics Simulation. Compos. Sci. Technol..

[22] Wang H., Jin K., Wang C., Guo X., Chen Z., Tao J. (2019). Effect of Fiber Surface Functionalization on Shear Behavior at Carbon Fiber/Epoxy Interface through Molecular Dynamics Analysis. Compos. Part A Appl. Sci. Manuf..

[23] Stoffels M.T., Staiger M.P., Bishop C.M. (2019). Reduced Interfacial Adhesion in Glass Fibre-Epoxy Composites Due to Water Absorption via Molecular Dynamics Simulations. Compos. Part A Appl. Sci. Manuf..

[24] Niuchi T., Koyanagi J., Inoue R., Kogo Y. (2017). Molecular Dynamics Study of the Interfacial Strength between Carbon Fiber and Phenolic Resin. Adv. Compos. Mater..

[25] Jiao W., Liu W., Yang F., Jiang L., Jiao W., Wang R. (2017). Improving the Interfacial Property of Carbon Fiber/Vinyl Ester Resin Composite by Grafting Modification of Sizing Agent on Carbon Fiber Surface. J. Mater. Sci..

[26] Li M., Liu H., Gu Y., Li Y., Zhang Z. (2014). Effects of Carbon Fiber Surface Characteristics on Interfacial Bonding of Epoxy Resin Composite Subjected to Hygrothermal Treatments. Appl. Surf. Sci..

[27] Zhang B., Shi F., Zhou Z., Yang J., Dai Z. (2009). Surface Characteristics of Carbon Fibers and Interfacial Properties of Carbon Fibers Reinforced BMI Matrix Composites. J. Wuhan Univ. Technol..

[28] Li Z., Liu L., Li J. (2021). Effect of pressure on structure of PAN-based carbon fibers at high temperature. J. Ceram..

[29] Li C. (2021). Study on the Time-Combination Effect on the Evolution of Graphite Crystalline of PAN-Based Carbon Fiber.

[30] Zhang M., Wang X., Zhou M., Zhai Z., Jiang B. (2020). The Effect of Self-Resistance Electric Heating on the Interfacial Behavior of Injection Molded Carbon Fiber/Polypropylene Composites through Molecular Dynamics Analysis. Polymer.

[31] Jiao W., Zheng T., Liu W., Jiao W., Wang R. (2019). Molecular Dynamics Simulations of the Effect of Sizing Agent on the Interface Property in Carbon Fiber Reinforced Vinyl Ester Resin Composite. Appl. Surf. Sci..

[32] Uematsu H., Mune K., Nishimura S., Koizumi K., Yamaguchi A., Sugihara S., Yamane M., Kawabe K., Ozaki Y., Tanoue S. (2022). Fracture Properties of Quasi-Isotropic Carbon-Fiber-Reinforced Polyamide 6 Laminates with Different Crystal Structure of Polyamide 6 Due to Surface Profiles of Carbon Fibers. Compos. Part A Appl. Sci. Manuf..

[33] Yang J., Zhai Z., Liu J., Weng C. (2021). Molecular Dynamics Simulation on the Adhesion Mechanism at polymer-mold Interface of microinjection Molding. J. Appl. Polym. Sci..

[34] LAMMPS Molecular Dynamics Simulation. Http://Lammps.Sandia.Gov/.

[35] Zhang H., Zhou Z., Qiu J., Chen P., Sun W. (2021). Defect Engineering of Carbon Nanotubes and Its Effect on Mechanical Properties of Carbon Nanotubes/Polymer Nanocomposites: A Molecular Dynamics Study. Compos. Commun..

[36] Jin Y., Duan F., Mu X. (2016). Functionalization Enhancement on Interfacial Shear Strength between Graphene and Polyethylene. Appl. Surf. Sci..

[37] Javan Nikkhah S., Moghbeli M.R., Hashemianzadeh S.M. (2015). Investigation of the Interface between Polyethylene and Functionalized Graphene: A Computer Simulation Study. Curr. Appl. Phys..

[38] Kiss P., Glinz J., Stadlbauer W., Burgstaller C., Archodoulaki V.-M. (2021). The Effect of Thermally Desized Carbon Fibre Reinforcement on the Flexural and Impact Properties of PA6, PPS and PEEK Composite Laminates: A Comparative Study. Compos. Part B Eng..

