# Supplementary material for: Molecular Dynamics Simulation on the Effect of Self-Resistance Electric Heating on Carbon Fiber Surface Chemical Properties and Fiber/PP Interfacial Behavior
Source: Polymers (Basel). 2022 Mar 5;14(5):1043. doi: 10.3390/polym14051043 (PMC8914934; doi:10.3390/polym14051043)
Supplement: Supplementary file 1 [file polymers-14-01043-s001.zip › polymers-1551241-supplementary.pdf]

Supporting Information

Molecular Dynamics Simulation on the effect of self-resistance electric heating on carbon fiber surface chemical properties and fiber/PP interfacial behavior

Qingzhu He [1], Jiaqing Liu [1], Muhan Zhang [1], Zhanyu Zhai [1, 2, *], Bingyan Jiang [1, 2]

[1] College of Mechanical and Electrical Engineering, Central South University, Changsha, 410083, China;

[2] State Key Laboratory of High Performance and Complex Manufacturing, Central South University, Changsha, 410083, China;

* Correspondence: zhanyuzhai@csu.edu.cn

Figure S1 shows the temperature field distribution of carbon fiber fabric under SRE heating with the current intensity of 12 A, 16 A, 20 A and 24 A. As seen, the electric current of 12 A, 16 A, 20 A and 24 A at steady stage corresponds the surface temperature of carbon fiber fabrics of 158±2.9℃, 212±3.2℃, 270±4.9℃ and 332±5.4℃ respectively, which has been added in the revised manuscript.

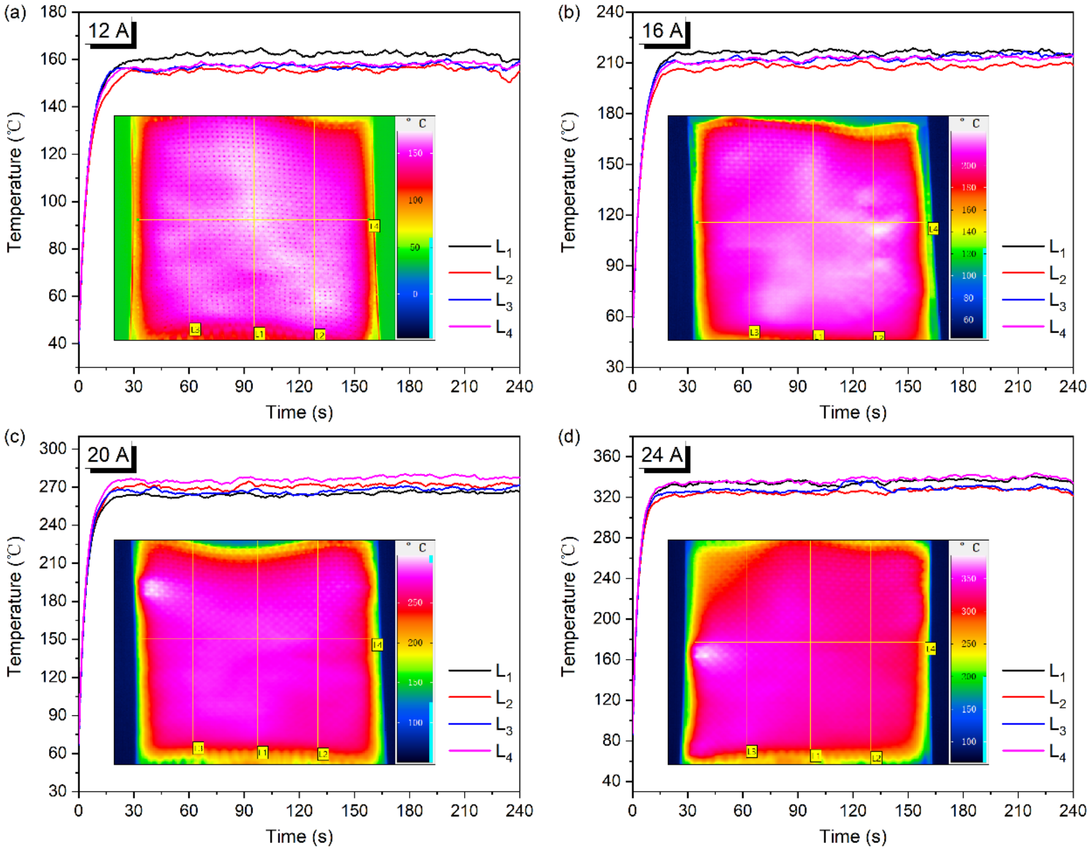

Figure S1. Temperature field distribution of carbon fiber fabric under SRE heating with different current intensity.
